# Prophylactic Sublingual Immunization with *Mycobacterium tuberculosis* Subunit Vaccine Incorporating the Natural Killer T Cell Agonist Alpha-Galactosylceramide Enhances Protective Immunity to Limit Pulmonary and Extra-Pulmonary Bacterial Burden in Mice

**DOI:** 10.3390/vaccines5040047

**Published:** 2017-12-06

**Authors:** Arshad Khan, Shailbala Singh, Gloria Galvan, Chinnaswamy Jagannath, K. Jagannadha Sastry

**Affiliations:** 1 Department of Pathology and Laboratory Medicine, The University of Texas Health Science Center, Houston, TX 77030, USA; Arshad.Khan@uth.tmc.edu (A.K.); Chinnaswamy.Jagannath@uth.tmc.edu (C.J.); 2 Department of Immunology, The University of Texas M.D. Anderson Cancer Center, Houston, TX 77030, USA; ssingh1@mdanderson.org (S.S.); GGalvan1@mdanderson.org (G.G.); 3 UTHealth Graduate School of Biomedical Sciences, The University of Texas MD Anderson Cancer Center, Houston, TX 77030, USA; 4 Department of Veterinary Sciences, The University of Texas M.D. Anderson Cancer Center, Bastrop, TX 77030, USA

**Keywords:** tuberculosis vaccine, mucosal immunity, sublingual vaccination, natural killer T cells, alpha-galactosylceramide, subunit vaccine, antigen 85B and ESAT-6, TH1 immune responses

## Abstract

Infection by *Mycobacterium tuberculosis* (Mtb) remains a major global concern and the available Bacillus Calmette-Guerin (BCG) vaccine is poorly efficacious in adults. Therefore, alternative vaccines and delivery strategies focusing on Mtb antigens and appropriate immune stimulating adjuvants are needed to induce protective immunity targeted to the lungs, the primary sites of infections and pathology. We present here evidence in support of mucosal vaccination by the sublingual route in mice using the subunit Mtb antigens Ag85B and ESAT-6 adjuvanted with the glycolipid alpha-galactosylceramide (α-GalCer), a potent natural killer T (NKT) cell agonist. Vaccinated animals exhibited strong antigen-specific CD4 and CD8 T cells responses in the spleen, cervical lymph nodes and lungs. In general, inclusion of the α-GalCer adjuvant significantly enhanced these responses that persisted over 50 days. Furthermore, aerosolized Mtb infection of vaccinated mice resulted in a significant reduction of bacterial load of the lungs and spleens as compared to levels seen in naïve controls or those vaccinated with subunit proteins, adjuvant , or BCG alone. The protection induced by the Mtb antigens and-GalCer vaccine through sublingual route correlated with a TH1-type immunity mediated by antigen-specific IFN-γ and IL-2 producing T cells.

## 1. Introduction

With nearly 2 billion infections worldwide and 8 million new diagnoses each year, *Mycobacterium tuberculosis* (Mtb) infections continue to pose major public health concerns [1,2,3,4,5]. Furthermore, the risk of developing tuberculosis (TB) is twenty times greater for HIV positive individuals and a third of illness and fatalities in HIV/AIDS patients are related to Mtb infections [6,7]. The currently available *M. bovis* strain Bacillus Calmette-Guerin (BCG) vaccine, although significantly reduces incidents in children, provides poor efficacy against pulmonary TB in adults [8,9]. Also, because BCG vaccine contains live bacteria, its administration poses a risk to HIV infected and other primary immunodeficient children [10]. Therefore, development of a TB vaccine that not only induces strong protective immunity but also provides better safety profile compared to BCG is a necessity. Based on their immuno-dominant nature, the secreted Mtb proteins Ag85B and ESAT-6 have been widely explored as safer candidate subunit vaccines against Mtb [11,12].

Primary immune response against Mtb depends on T helper type-1 (Th1) cells producing IFN-γ and TNF-α, that can activate the macrophages of the granulomas to control the growth of Mtb [13,14]. Since transmission and establishment of infection with Mtb in cases of pulmonary disease occurs by inhalation through the respiratory route, mucosal immune responses are the first line of defense. Mucosal vaccination offers the unique advantage of administering vaccines by the most practical nasal and oral/sublingual routes for eliciting effective immune responses at local as well as distant sites that are relevant for protection against mucosal pathogens such as influenza virus and Mtb in the lungs, and HIV and HPV in genital tissues [15,16,17,18]. Among the various mucosal routes for delivery of vaccines explored, sublingual immunization has the advantage of being a practical and needle-free inexpensive option for largescale vaccination even in resource-limited areas [16,19]. In addition, unlike intranasal route, sublingual immunization has a safety advantage with no risk of retrograde transport of antigen and/or adjuvant from vaccine formulation to the brain and other neural tissues [20]. However, due to the inherent tolerance in the mucosal tissues induction of vaccine-specific immune responses requires inclusion of adjuvants that activate innate immune modulators to increase the immunogenicity of co-administered antigens. 

The synthetic glycolipid, alpha-galactosylceramide (α-GalCer) has a well-established safety and efficacy profile as an adjuvant for a variety of antigens [19,21,22,23]. This is because α-GalCer serves as a potent activator of invariant natural killer T (iNKT) cells, a major innate cell type that can jump-start the adaptive immunity by activating dendritic cells for efficient antigen presentation [24,25]. Several studies in the literature as well as from our group demonstrated the effectiveness of α-GalCer as an adjuvant when delivered by mucosal routes to elicit adaptive immune responses to co-administered antigens at multiple mucosal sites [19,24,26,27,28,29]. Sublingual route for prime-boost immunization regimen employing α-GalCer adjuvant can induce strong and broadly disseminated antigen-specific CD4^+^ and CD8^+^ T lymphocyte responses, specifically in the lungs and lung-draining lymph nodes (DLN) that are most relevant for pulmonary immunity [19].

In the current study, we tested the effectiveness of a sublingual prophylactic vaccine constituting Mtb derived Ag85B and ESAT-6 proteins along with the α-GalCer adjuvant. Analysis of T lymphocyte activation in the spleen and lungs of immunized mice demonstrated the ability of the subunit vaccine to induce antigen specific IFN-γ and IL-2 responses by CD4 T cells and IFN-γ response by CD8 T cells. Furthermore, the vaccination approach generated stable and persistent cell mediated immunity that was effective at limiting bacterial burden in lungs and spleens of mice after aerosol challenge with Mtb. 

## 2. Materials and Methods

### 2.1. Mice 

Female C57Bl/6 mice aged 6–10 weeks were purchased from the National Cancer Institute (Frederick, MD, USA) or Jackson Laboratory (Bar Harbor, ME, USA). The animals were maintained in specific pathogen-free environment at the institutional animal facilities. Studies evaluating the immunogenicity of the vaccine in mice were conducted at The UT MD Anderson Cancer Center (Houston, TX, USA) and *in vivo* Mtb challenge experiments were conducted at The University of Texas McGovern Medical School, Houston. Both the animal facilities are fully accredited by the Association for Assessment and Accreditation of Laboratory Animals Care International. All animal procedures were conducted in compliance with the institutional animal and bio-safety approved protocols. In general, the experiments were performed using 5 mice/group and repeated 2 times.

### 2.2. Reagents

Recombinant Mtb proteins Ag85B and ESAT-6 were obtained from BEI Resources (Manassas, VA, USA), and dissolved in 1X PBS at a concentration of 5 mg/mL. The alpha-galactosylceramide (α-GalCer) was procured from Diagnocine LLC (Hackensack, NJ, USA) and dissolved in dimethyl sulfoxide, (Sigma, St. Louis, MO, USA) at a concentration of 1 mg/mL.

### 2.3. Immunization

Mice were first anesthetized by i.p. administration of ketamine (100 mg/kg) and xylazine hydrochloride (10 mg/kg) and then immunized by sublingual route according to the protocol established in the laboratory [19]. To avoid inadvertent swallowing, the total volume of inoculum was limited to 7 μL/animal and the animals were maintained with their heads in ante-flexion untill they regained consciousness. Each animal was immunized with a mixture of Ag85B (10 μg) and ESAT-6 (10 μg) either with or without α-GalCer (2 μg). The Doses of antigens and α-GalCer were selected as per their effective immunogenic concentration reported in earlier animal studies [11,12,27,29]. Mice were immunized two times at 7 day interval and were sacrificed at days 14 or 56 after the first immunization to determine effector and persistent T lymphocyte responses, respectively. For the comparison with BCG vaccine, a group of mice were vaccinated with single dose of *M. bovis* BCG (Pasteur strain) (10^6^ CFU per dose) via subcutaneous route. Antigen-specific T cell responses were evaluated in the spleens, lungs and vaccine draining cervical lymph nodes. 

### 2.4. IFN-γ ELISpot Assay

The IFN-γ ELISpot assay kit (BD Biosciences, San Jose, CA, USA) was used to determine Ag85B and ESAT-6 specific responses of cells isolated from spleens, lungs and cervical lymph nodes using a previously described protocol [23]. Aliquots (2 × 10^5^) of cells from the different tissues were cultured in duplicate with either Ag85B (5 μg/mL), ESAT-6 (5 μg/mL), or Concanavalin A (5 μg/mL) for 48 h at 37 °C and 5% CO_2_. Enumeration of spots representing individual cells producing IFN-γ was conducted by Zellnet Consulting Inc., Fort Lee, NJ, USA) using KS-ELISPOT automatic system (Carl Zeiss Inc., Thornwood, NY, USA). Responses were considered positive only when they were above 10 Spot forming Cells (SFC)/2 × 10^5^ input cells and at least twice the number obtained in cells cultured with medium alone. 

### 2.5. Intracellular Cytokine Production Assay

The antigen specific responses of CD4 and CD8 T cells were determined by performing an intracellular cytokine production assay. Cells from spleens and lungs of immunized mice were stimulated ex vivo with either Ag85B (5 μg/mL) or ESAT-6 (5 μg/mL) overnight at 37 °C and 5% CO_2_ followed by incubation of the cells with GolgiPlug reagent (BD Biosciences, San Jose, CA) in complete medium for 6 h prior to flow cytometry. Cells incubated with PMA (2.5 ng/mL) and Ionomycin (2.5 ng/mL) served as positive control. The samples were first stained with Pacific Blue-conjugated anti-CD3 (clone 500A2), PerCP Cy5.5 conjugated anti-CD8 (clone 53-6.7), FITC conjugated anti-CD4 (clone RM4-5) and PE-Cy7 conjugated anti-CD107a (clone 1D4B) monoclonal Antibodies (mAbs). The cells were then permeabilized for intracellular cytokine staining with PE-conjugated anti-IFN-γ (clone XMG1.2) and APC Cy7 conjugated anti-IL2 (clone JES6-5H4) mAbs in 1× Perm/Wash Buffer (BD Biosciences). All the mAbs were purchased from BD Biosciences (San Jose, CA, USA). Data were acquired on LSRII flow cytometer and analyzed using FlowJo software (Tree Star Inc, Ashland, OR, USA). Lymphocytes were gated using the forward scatter and side scatter plots followed by gating of T lymphocytes using side scatter and CD3. The levels of each cytokine were determined by subtracting out background values from cells cultured with medium only. Fluorescence minus one (FMO) stains were also used as negative controls 

### 2.6. Mycobacterium Tuberculosis Challenge

For determining the prophylactic efficacy of the vaccine, different groups of C57Bl/6 mice (6–8 week old) were immunized as described above by sublingual route twice at weekly interval with a mixture of the recombinant Mtb proteins Ag85B (10 μg) and ESAT-6 (10 μg) in the presence or absence of α-GalCer (2 μg). Control groups received α-GalCer (2 μg) alone or PBS. An additional group of mice immunized once by subcutaneous route with BCG (10^6^ CFU/ mouse) 21 days prior to challenge was used for comparing the protective efficacy of the sublingual Mtb subunit vaccine. Three weeks after final immunization, the animals were infected with *Mycobacterium tuberculosis* Erdman (ATCC 35801) by the aerosol route (100 CFU/mouse through Glas-Col apparatus) as described previously [30]. Four weeks later the animals were sacrificed and viable bacteria in the lungs and spleens were enumerated as described in the literature [31]. Briefly, the protocol included plating 10-fold serial dilutions of organ homogenates onto 7H11 agar plates (# 37 Hardy Diagnostics, Santa Maria, CA, USA) and colonies were counted after an incubation period of 3 weeks. Lymphocytes from the lungs and spleens from a separate set of immunized and challenged mice were also isolated and intracellular cytokine staining for IFN-γ was performed on ex vivo stimulated cells to evaluate the antigen specific responses correlating with protective efficacy of the different vaccinations. 

### 2.7. Statistical Analysis

The immune responses and bacterial loads were expressed as averages of 3–6 mice/group. Paired two-tailed Student’s *t*-test/one way ANOVA with post-hoc analysis was used to determine the significance between different immunization groups. All analyses were performed using GraphPad Prism, version 6 (GraphPad Software, San Diego, CA, USA) and *p* ≤ 0.05 was considered statistically significant. 

## 3. Results

### 3.1. Immunogenicity of Sublingual Mtb Subunit Vaccination

**Figure 1:**
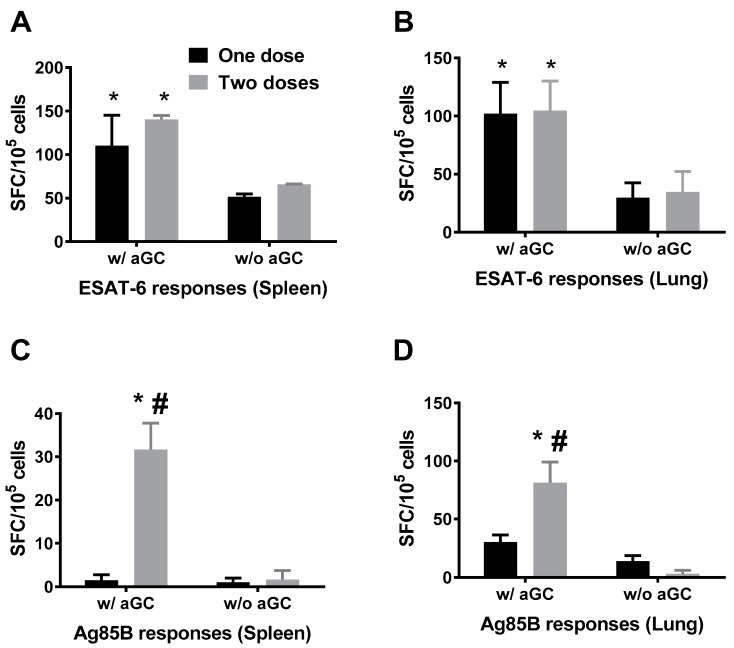
Sublingual immunization with *Mycobacterium tuberculosis* (Mtb) proteins Ag85B and ESAT-6 in the presence of alpha-galactosylceramide (α-GalCer) adjuvant induces systemic and mucosal T cell responses. Separate groups of mice (*n* = 4–6) were immunized by sublingual route with one or two doses of mixture of Mtb proteins Ag85B and ESAT-6 in the presence or absence of α-GalCer adjuvant (w/aGC and w/o aGC, respectively) at 7 day intervals and sacrificed 7 days after each immunization to determine immune responses. Single cell suspensions from spleen and lung tissues were analyzed for IFN-γ producing cells specific for ESAT-6 (Panels (**A**,**B**)) and Ag85B (Panels (**C**,**D**)) using a standard IFN-γ ELISpot assay. Responses specific to the Mtb proteins were determined by subtracting the background values of medium stimulation from that of the individual protein stimulation and the mean ± S.D values were expressed as spot forming cells (SFC) per 10^5^ input cells. Significant responses (*p* < 0.05) between groups vaccinated in the presence and absence of α-GalCer (*) and between one and two doses (#) of vaccine delivered were noted. The experiment was performed using 5 mice/group and repeated 2 times.

The immunogenicity of sublingually administered vaccine consisting of Mtb proteins Ag85B and ESAT-6 along with α-GalCer adjuvant was determined by immunizing mice twice at 7 day interval and sacrificing 7 days after each immunization. Control mice were immunized with Mtb proteins in the absence of α-GalCer adjuvant. The breadth of Mtb antigen specific T cell immune responses in the spleens and lungs were evaluated by the IFN-γ ELISpot and intracellular cytokine production assays. Data from the ELISpot analyses demonstrated that vaccination with the Mtb proteins in the presence of α-GalCer adjuvant induced significantly higher T cell responses relative to administering the proteins in the absence of the adjuvant. Furthermore, a single sublingual immunization was effective in inducing significant IFN-γ producing cells specific to ESAT-6 in the lungs and spleens of animals immunized with the Mtb proteins in the presence, but not absence, of the α-GalCer adjuvant (Figure 1A,B). In contrast, significant responses to the Ag85B antigen were observed only after delivering two doses of the vaccine in the spleen as well as lung (Figure 1C,D). 

**Figure 2:**
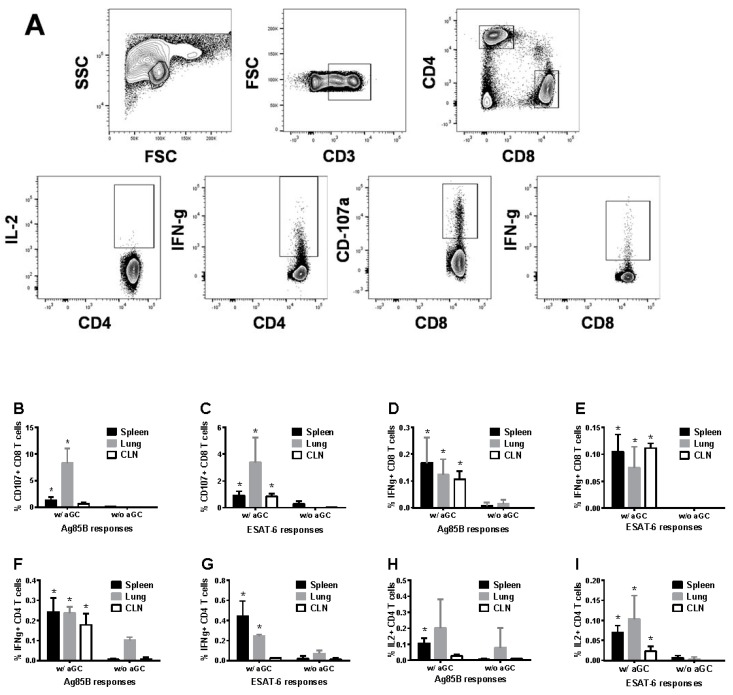
Sublingual immunization with Mtb proteins Ag85B and ESAT-6 in the presence of α-GalCer adjuvant induces CD4 and CD8 T cell responses. C57BL/6 female mice were immunized as in Figure 1 and cells isolated from lung, spleen and cervical lymph node(CLN) were analyzed by intracellular cytokine flow cytometry for CD8 and CD4 T cell responses specific to the Mtb proteins Ag85B and ESAT-6. Flow cytometry gating strategy for detecting the different lymphocyte subsets expressing the cytokines analyzed (panel (**A**)). Data for the CD8 T cells positive for CD107a and IFN-γ (panels (**B**–**E**)), and CD4 T cell for IFN-γ and IL2 (panels (**F**–**I**)). Significant responses (*, *p* < 0.05) between groups vaccinated in the presence and absence of α-GalCer (w/aGC and w/o aGC, respectively) were noted. The experiment was performed using 5 mice/group and repeated 2 times.

Intracellular cytokine analyses were performed to determine CD4 and CD8 T cell responses specific to the Mtb proteins Ag85B and ESAT-6, and the gating strategy for the flow cytometry analyses is shown in Figure 2A. We observed CD8 T cells positive for the cytotoxic degranulation marker CD107a (Figure 2B,C) or producing IFN-γ (Figure 2D,E) as well as helper CD4 T cells producing IFN-γ (Figure 2F,G) or IL-2 (Figure 2H,I) in the spleens and lungs of the mice immunized by sublingual route with the recombinant Mtb proteins. These responses were significantly higher only when α-GalCer adjuvant was included in the vaccination. A simultaneous examination of NKT cells was not done here since our earlier studies using this immunization regimen showed activation of NKT cells upon sublingual α-GalCer administration [26]. 

### 3.2. Persistence of Immunity from Sublingual Vaccination with Mtb Proteins and α-GalCer Adjuvant

**Figure 3:**
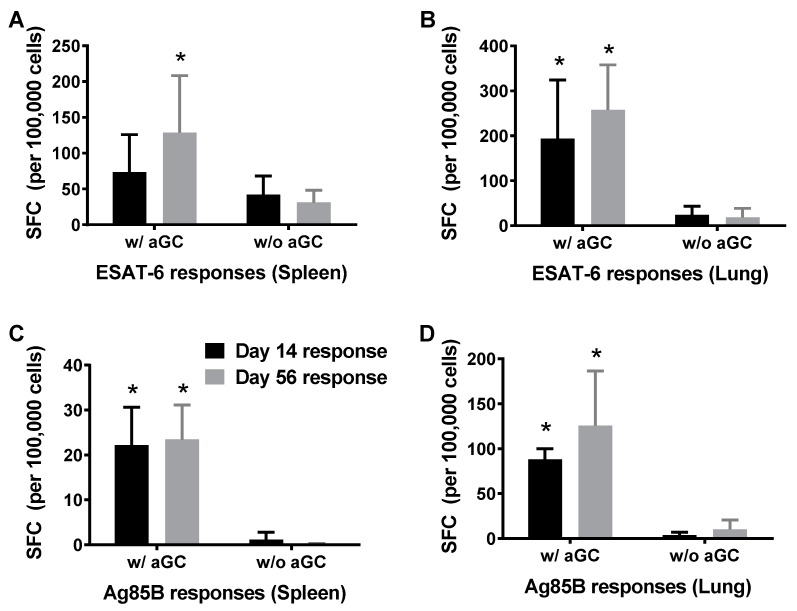
Induction and persistence of antigen-specific immunity after sublingual immunization with Mtb proteins Ag85B and ESAT-6 in the presence of α-GalCer adjuvant. C57BL/6 female mice were immunized as in Figure 1 by the sublingual route and sacrificed on days 14 and 60 for determining the effector and long term persistent T cell responses, respectively. Cells isolated from spleen and lung were analyzed for IFN-γ producing cells specific for ESAT-6 (Panels (**A**,**B**)) and Ag85B (Panels (**C**,**D**)) using a standard IFN-γ ELISpot assay. Responses specific to the Mtb proteins were determined by subtracting the background values of medium stimulation from that of the individual protein stimulation and the mean ± S.D values were expressed as spot forming cells (SFC) per 10^5^ input cells. See methods section for details and cut-off values for positive responses. Significant responses (*, *p* < 0.05) between groups vaccinated in the presence and absence of α-GalCer (w/aGC and w/o aGC, respectively) were noted. The experiment was performed using 5 mice/group and repeated 2 times.

We determined whether sublingual vaccination with the Mtb proteins Ag85B and ESAT-6 in the presence and absence of the α-GalCer adjuvant induced longer lasting immunity in both mucosal and systemic tissues. Different groups of mice were immunized twice at 7-d intervals and one sub group of mice was sacrificed 7 days after second immunization for determining effector response and remaining mice were sacrificed at day 56 post immunization to evaluate the persistence of immunity in the spleen and lung tissues by IFN-γ ELISPot assay. The results obtained demonstrated the induction and persistence of immune responses specific to both ESAT-6 (Figure 3A,B) and Ag85B (Figure 3C,D) that were significantly higher when the Mtb proteins were administered in the presence of α-GalCer. 

### 3.3. Sublingual Vaccination with Mtb Proteins and α-GalCer Adjuvant Affords Protection against Lung Mtb Challenge

**Figure 4:**
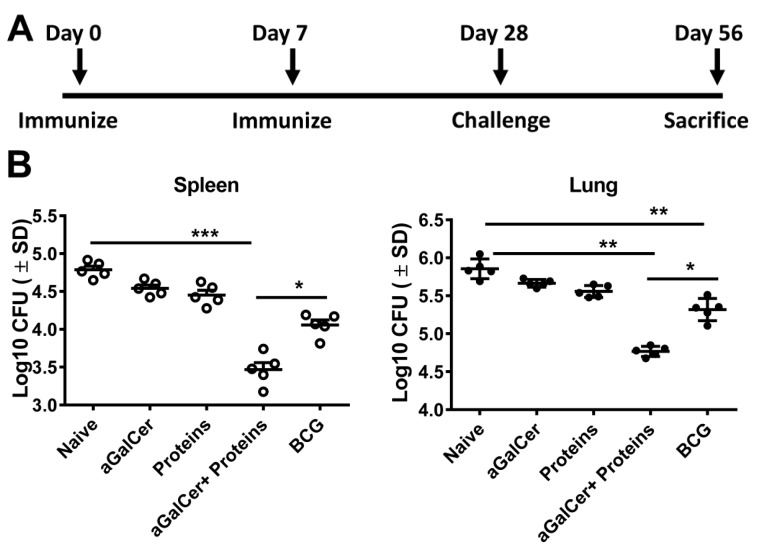
Sublingual immunization with Mtb proteins Ag85B and ESAT-6 in the presence of α-GalCer adjuvant significantly reduces pulmonary and extra-pulmonary bacterial load: C57BL/6 female mice were immunized by the sublingual route twice at 7 day intervals with a mixture of the recombinant Mtb proteins Ag85B and ESAT-6 in the presence and absence of the adjuvant α-GalCer (α-GalCer + Proteins and Proteins, respectively) or with adjuvant alone (α-GalCer) or Bacillus Calmette-Guerin (BCG) or untreated (naïve) and challenged on day 28 with Mtb by aerosol. (Panel (**A**)): The bacterial burden in the spleens (Panel (**B**), left) and lungs (Panel (**B**), right) of mice in the different groups were analyzed on day 56 (28 days post challenge) as described in the methods section. Significantly lower (**, *p* < 0.005) bacterial burden in the spleens of mice vaccinated with either BCG or the Mtb proteins in the presence of α-GalCer adjuvant were observed when compared to naïve mice. Similar analysis in the lungs also showed significantly lower (***, *p* < 0.0005) bacterial burden in mice vaccinated with the Mtb proteins in the presence of α-GalCer adjuvant when compared to naïve mice. In both spleens and lungs of mice vaccinated with Mtb proteins in the presence of α-GalCer adjuvant the bacterial burden was significantly lower (*, *p* < 0.05) than those in mice immunized with BCG. Data shown are from one of two separate experiments each with groups of 5 mice.

We tested the prophylactic efficacy of the Mtb subunit vaccine using separate groups of mice immunized twice at a weekly interval with either α-GalCer alone, Mtb proteins alone, or Mtb proteins along with α-GalCer (Figure 4A). Mice receiving single subcutaneous immunization with BCG were used as positive control for prophylaxis. On day 28 post first immunization, mice were challenged with *Mycobacterium tuberculosis* Erdman (ATCC 35801) by aerosol route and were sacrificed on day 56 (i.e., 28 days post challenge) for evaluating bacterial burden and cell mediated immune responses in lungs and spleens. As shown in Figure 4B, sublingual vaccination with the Mtb proteins Ag85B and ESAT-6 in the presence of the α-GalCer adjuvant resulted in significant protection in terms of reduced bacterial burden in lungs as well as spleens, relative to naive controls or other groups of mice immunized with either α-GalCer alone or Mtb proteins alone (ANOVA analyses with Bonferroni’s multiple comparisons test). The BCG vaccine also significantly reduced lung bacterial burden relative to naïve mice and those receiving α-GalCer adjuvant alone but not the Mtb proteins either with or without α-GalCer adjuvant. Furthermore, we observed that the bacterial burden in the lungs as well as spleens after sublingual immunization with the Mtb protein subunit vaccine (containing the α-GalCer adjuvant) was significantly lower relative to that in the mice receiving the BCG vaccine.

### 3.4. Sublingual Vaccination Induces T Cell Responses which Correlate with Protection against Lung Mtb Challenge

**Figure 5:**
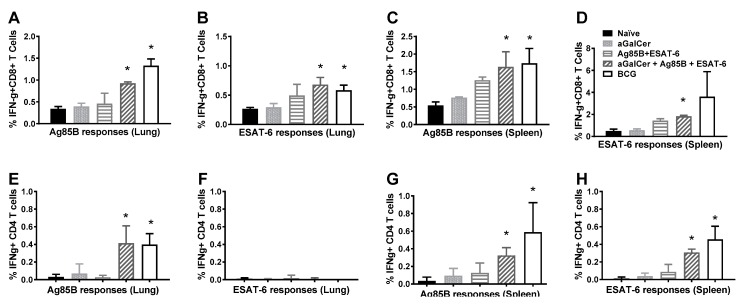
Sublingual immunization with Mtb proteins Ag85B and ESAT-6 in the presence of α-GalCer adjuvant affords protection against lung Mtb challenge through inducing antigen-specific CD4 and CD8 T cell responses. C57BL/6 female mice were immunized by the sublingual route as in Figure 1 and challenged on day 28 with Mtb by aerosol. Cells isolated from the lung and spleen tissues were analyzed on day 56 (28 days post challenge) by intracellular cytokine flow cytometry for IFN-γ producing CD8 (Panels (**A**–**D**)) and CD4 T (Panels (**E**–**H**)) cells in response to stimulation with the Mtb proteins Ag85B and ESAT-6. Significance (*, *p* < 0.05) was calculated by ANOVA analyses for comparing values between naïve animals and those vaccinated with the combination of the Mtb proteins plus α-GalCer or the BCG. The experiment was performed using 5 mice/group.

Intracellular cytokine flow cytometric analysis was performed with lymphocytes isolated from spleens and lungs of mice after sublingual immunization with the Mtb subunit vaccine followed by Mtb challenge (Figure 5). The results obtained demonstrated significantly higher frequencies of antigen-specific IFN-γ^+^ CD8 T cells in the lungs (Figure 5A,B) as well as spleen (Figure 5C,D), relative to those in naïve animals or animals immunized with α-GalCer alone. These responses in Mtb subunit vaccinated mice were similar in magnitude to those in mice receiving the BCG vaccine. We observed significantly higher percentages of CD4 T cells producing IFN-γ in response to Ag85B in the lung and spleen (Figure 5E,G) of mice challenged with Mtb after receiving the subunit Mtb vaccine, relative to the control groups. The IFN-γ^+^ CD4 T cell responses specific to ESAT-6 in the spleen were significantly higher in the subunit vaccinated mice (Figure 5H). Thus, both CD4 and CD8 T cells producing IFN-γ in response to Ag85B were observed in the lung as well as spleen tissues of mice experiencing significant protection from Mtb challenge, in terms of reduced lung bacterial loads, suggesting the potential association of Ag85B specific T cell immunity with protective efficacy. 

## 4. Discussion

Limited or variable efficacy of BCG in adults against pulmonary TB has necessitated newer approaches to design an effective TB vaccine using the most potent antigens and adjuvants to boost immune responses and an optimal vaccination routes.. Glycolipid, alpha-galactosylceramide (α-GalCer) is known to rapidly activate murine and human NKT cells and its role in modulating T cell response is also well recognized [32,33]. When combined with Isoniazid, synergistic effect of α-GalCer as a therapeutic agent in controlling Mtb infection has been demonstrated in an earlier study [34]. However, adjuvant capacity of α-GalCer in boosting immunogenicity of Mtb antigens remained unexplored. Here we have shown that by using the antigen Ag85B and ESAT-6 the immunogenicity of the antigens could be enhanced when α-GalCer, a potent activator of NKT cells, was delivered concurrently (Figure 1). Interestingly, ESAT-6 and Ag85B behaved differently in terms of their dependency on doses to elicit the antigen specific immune response. Unlike ESAT-6, immune response induced by Ag85B was dependent on boosting, which perhaps could be attributed to the difference in the stability of these proteins while transitioning through mucosal membranes. ESAT-6 has been known to trigger detrimental effect on overall immune response due to induction of type 1 interferon by this protein [35,36]. However, another study has also indicated that ESX-1-mediated secretion is required for the production of host type I IFNs by ESAT-6 during infection in vivo and in macrophages in vitro [37] . Since we have here used only protein as subunit vaccine without ESX-1 secretion system, we believe that ESAT-6 protein alone may not have any suppressive effect on the immune response. Mice immunized with a combination of α-GalCer and ESAT-6/Ag85B induced 2–3 fold higher numbers of IFN-γ producing antigen specific T cells in both spleens and lungs, relative to immunizing with the proteins in the absence of α-GalCer supporting an important role for the NKT cell agonist in priming adaptive immune responses. Although, the mechanism through which NKT cells positively influence the priming of T cells through antigen presentation is not completely clear, others have indicated that stimulation of NKT cells can lead to trans-activation of a variety of innate and adaptive immune system cells, including conventional T cells and dendritic cells [38,39]. While NKT cells could shift the balance towards Th2 response in the case of autoimmunity, they can also tilt it in favor of Th1 response during infections [40]. The role of α-GalCer in promoting the production of Th1 cytokines was clearly evident during Mtb infection in the present study (Figure 2 and Figure 5). Given the substantial evidence for the contribution of NKT cells in combating Mtb infection, pharmacological agents like α-GalCer that target NKT cells could be an attractive avenue of adjuvant discovery for TB vaccines [41,42]. 

The potency of α-GalCer delivery via sublingual route to enhance immunity against tumors as well as intracellular infections such as hepatitis and malaria has been shown in earlier studies [43,44,45]. By sublingual delivery, as opposed to oral gavage, the antigens are absorbed directly into the bloodstream from oral mucosa with limited proteolytic degradation and without induction of anaphylaxis [46]. Since the primary site of disease manifestation of TB is the lungs, selection of mucosal route of administration of vaccine and adjuvant the advantage of activating local NKT cells as well in the circulating population. In this study, induction of CD4 and CD8 T cells response was seen in lungs as well as secondary lymphoid organs when α-GalCer was combined with the vaccine (Figure 2 and Figure 5). The data thus suggests that sublingually delivered α-GalCer could enhance the cell-mediated immunity to the co-administered TB antigens at local lung lesions. Although, equivalent or higher level of IFN γ producing ESAT-6/Ag85B specific CD4 and CD8 T cells were seen in BCG vaccinated mice as compared to mice vaccinated with α-GalCer and Ag85B + ESAT-6, latter group of vaccinated mice provided better protection (Figure 4 and Figure 5). This confirms the contribution of NKT cells in protection against *Mtb* apart from IFN-γ producing antigen specific CD4 and CD8 T cells. Indeed, IFN-γ independent contribution of NKT cells in the protection against *Mtb* in vitro and in vivo has been reported by others as well [47,48]. Mechanistically, activation of NK T cells could significantly suppress the growth of intracellular *Mtb* in macrophages [48]. Interestingly, NKT cell mediated control of *M. tuberculosis* growth was found to be CD1d-dependent, and did not require IFN-γ or any other Th1 cytokines such as TNF-α, IL-2 and IL-18. This study also demonstrated that production of GM-CSF by NKT cells played a key role in their antimicrobial effector function, further implicating the role for GM-CSF in inducing T cell immunity against *M. tuberculosis*. Considering the distribution of NKT cells at mucosal sites and their known contribution in pulmonary immunity [47,49], targeting this subpopulation of T cells could be an attractive approach to improve the efficacy of TB vaccine. Improved quality of CD4 and CD8 T cell responses was also found to be associated with enhanced protection against Mtb challenge in mice that were adjuvanted with α-GalCer compared to unadjuvanted or BCG vaccinated mice in our study, confirming the role of T cells in combating TB (Figure 2 and Figure 4). Adjuvant effect of a-GalCer on subunit vaccine for improved bacterial clearance after Mtb challenge was not only seen at pulmonary sites but also at extra-pulmonary sites (e.g., spleen in the current study) of infected mice.

Results from this study also demonstrated that the sublingual vaccination regimen could not only induce Mtb antigen-specific effector CD4/CD8 T cell responses but also extend their persistence to nearly 60 days, suggesting that α-GalCer adjuvant promotes long term immunity against Mtb infection (Figure 3). Our results are consistent with reports in the literature that suggested selective bystander proliferation of persisting CD4^+^ and CD8^+^ T cells upon NKT cell activation through IFN-γ or IL-12-dependent pathway [50]. Failure to induce substantial central memory T cells is one of the reasons cited for poor efficacy of BCG vaccine against adult TB [51]. Our results thus indicate α-GalCer, a potent NKT cell activator could provide long term protection against TB. It appears important to examine the different subsets of persistent memory T cells induced during NKT cell activation and, whether such an approach could enhance the potency of attenuated BCG strains and other candidate vaccines of Mtb. Additionally, these results also advocate exploring the potential of sublingual administration of α-GalCer in boosting the efficacy of BCG. Altogether, the finding presented in this study support the adjuvant potential role of α-GalCer and of the sublingual mucosal route of vaccination to improve protective efficacy of vaccines against Mtb infections. 

## 5. Conclusions

In summary, the evidences presented here demonstrate that sublingual administration of α-GalCer, combined with Mtb antigens Ag85B and ESAT-6, provide significantly higher level of protective immunity in mice than standard BCG vaccination against an aerosol challenge with Mtb. The results thus provide the basis for critical role of NKT cell mediated immunity in effective control of Mtb infections.
